# The Affect Heuristic and Risk Perception – Stability Across Elicitation Methods and Individual Cognitive Abilities

**DOI:** 10.3389/fpsyg.2020.00970

**Published:** 2020-06-12

**Authors:** Kenny Skagerlund, Mattias Forsblad, Paul Slovic, Daniel Västfjäll

**Affiliations:** ^1^Department of Behavioural Sciences and Learning, JEDI-Lab, Linköping University, Campus Valla, Linköping, Sweden; ^2^Decision Research, Eugene, OR, United States; ^3^Department of Psychology, University of Oregon, Eugene, OR, United States

**Keywords:** affect heuristic, cognitive reflection, risk perception, decision making, risk

## Abstract

The reliance on feelings when judging risks and benefits is one of the most fundamental valuation processes in risk perception. Although previous research suggests that the affect heuristic reliably predicts an inverse correlation between risk and benefit judgments, it has not yet been tested if the affect heuristic is sensitive to elicitation method effects (joint/separate evaluation) and to what extent individual differences in cognitive abilities may mediate the risk–benefit correlation. Across two studies we find that (1) the risk–benefit correlation is stable across different elicitation methods and for different domains (e.g., social domain, sensation-seeking domain, health domain, economic domain) and (2) the strength of the inverse correlation is tied to individual cognitive abilities—primarily cognitive reflection ability.

## Introduction

For a long time, the general view was that human decision making was a matter of rational, cognitive processing in which alternatives were exhaustively explored and calculated upon (Västfjäll and Slovic, 2013). However, recent developments during the past decades have led researches to increasingly acknowledge the role that affective states play in human decision making (Loewenstein et al., 2001; Västfjäll et al., 2016). This has led to the development of various forms of dual-process theories (e.g., Sloman, 1996; Stanovich and West, 2000) of decision making. Several researchers suggest that there is an interaction between more affective, experiential systems and deliberative systems (labeled System 1: fast thinking and System 2: slow thinking, respectively; Kahneman, 2011). One of the characteristics of experiential thinking is its affective basis. Although deliberative analyses are certainly important in many decision-making circumstances, reliance on affect and emotion as sources of information tends to be a quicker, easier, and more efficient way to navigate in a complex, uncertain, and sometimes dangerous world (Schwarz and Clore, 1988). Many theorists have given affect a direct and primary role in decision making (Damasio, 1994; Loewenstein and Lerner, 2003). One domain of human decision making that seems to be clearly entangled with affective processes is the realm of human risk judgment. Risk has been suggested to be perceived and acted upon in two ways: (1) risk-as-analysis, in which risk judgments are driven by logical reasoning and deliberation and (2) risk-as-feelings, in which judgments of risk are the result of momentary and intuitive reactions to averse events and dangers (Loewenstein et al., 2001; Slovic and Peters, 2006). Mounting evidence suggests that human evaluation of risk is driven by affective states, which has been attributed to the fact that human beings exploit the so-called *affect heuristic* (Slovic et al., 2002) ubiquitously in judgment and decision making. The affect heuristic refers to the fact that people make judgments based on representations of objects or events that are marked with valenced affect. By consulting the affective impression with which something is tagged instead of doing laborious calculations and utility maximizations, one can save time and effort that works sufficiently well in many situations for both humans (Slovic et al., 2004, 2007) and animals (Kralik et al., 2012). Empirical support for this claim was first established by Alhakami and Slovic (1994) when they discovered an inverse relationship between judgments of risks and benefits of various everyday activities and technologies. This is noteworthy given that objective evaluations of risk and benefits of activities and events in the world often should be independent of one another or even positively correlated (Slovic, 1987). For example, nuclear power should be deemed to be both risky and beneficial. In addition, Alhakami and Slovic (1994) found that the strength of the perceived benefit was linked to the estimated level of risk involved, suggesting that what people *feel* about the activity drives the judgments. A causal link between judgments of risk and benefit was established by Finucane et al. (2000), where they manipulated the amount of information given to the participants about various scenarios. By giving more favorable information about a certain activity, the affective evaluation increased. This manipulation led the participants to judge the activities to be more beneficial while simultaneously lowering the judgments of risk (Finucane et al., 2000). In the same vein, a study by Keller et al. (2006) found that evoking negative affect resulted in an increased level of perceived risks, which also has been linked to the possibility that the affect heuristic may lead to biased decisions when risk is a factor (Siegrist and Sutterlin, 2014). Similarly, Västfjäll et al. (2014) found that incidental negative affect amplified reliance on the affect heuristic leading to stronger inverse correlations between risk and benefits of a set of everyday behaviors. Further support for the affect heuristic came from a second experiment by Finucane et al. (2000) showing that the inverse relationship between perceived risks and benefits increased under time pressure when opportunity for analytic deliberation was reduced. These findings corroborate the notion that affective information underlies judgments of risk and benefit, but also confirm that there is a causal link between perceptions of risk and benefits of a given activity. A novel way of looking into the affective component of risk perception was also developed by Dohle et al. (2010), who established this link by using a version of the implicit association test, thus verifying the stability of the link between risk perception and affect beyond correlations of explicit self-reported ratings (see also Townsend et al., 2014). A longitudinal study also examined the stability of the risk and benefit judgments (Connor and Siegrist, 2016). The authors assessed risk and benefit judgments at two time points, and the follow-up assessment was made after 2 years had elapsed after the initial assessment. They found that risk and benefit judgments were moderately stable and that participants likely relied on the affect heuristic (Connor and Siegrist, 2016).

Still, one caveat about the empirical findings that have established an inverse correlation between judgments of risk and benefit pertains to the fact that these evaluations are done simultaneously. Research has shown that people make different evaluations about preferences depending on whether the options are presented in isolation (separate evaluation) or side-by-side (joint evaluation), sometimes resulting in preference reversals (Hsee, 1996; Hsee et al., 1999). Preference reversals have been suggested to be driven by the relative ease with which one evaluates the different options (i.e., evaluability). The rationale is that the value of a given alternative, which may be difficult to quantify, is more readily materialized when presented with a second alternative allowing for direct comparison. Given that the judgments of relative risk of various activities and domains often lack a clear baseline and metric as a reference frame, it is reasonable to assume that risk and benefit judgments are weak in evaluability (unless infused with easily evaluable affective meaning). If so, the apparent inverse correlation could be attributed to the joint mode in which these activities were evaluated. No study has, to date, verified that separate evaluations of risk and benefits show the same pattern as joint evaluations. A recent set of studies by Frey et al. (2017) showed that risk judgments differ depending on the method used to elicit them. Furthermore, a recent study by Kusev et al. (2020) showed that respondents’ risk preferences depended on the available choice options. The authors argue that the risk preferences are constructed “on the fly” during risk elicitation and that preferences are inherently unstable for any given individual. Thus, risk preferences are sensitive to context and choice options (Kusev et al., 2020). From the standpoint of the affect heuristic, one possible mechanism is that the affect heuristic is invoked in the context of any choice options or risk framing and, thus, informs judgments of risk and benefits despite otherwise variable risk behavior. Therefore, one aim of the current research project is to establish whether the inverse relationship can be found in both a joint condition and a separate condition and, thus, displays stability across elicitation methods. If so, we would strengthen the assumption that an affect heuristic drives the judgments of both risk and benefits. The second aim of this project is to investigate another form of stability: across methods of assessing/inducing reliance on System 1 versus System 2 processing. Given that Finucane et al. (2000) found that the inverse correlation increased under time pressure (a situational manipulation), it is important to examine whether individual differences in reliance on System 1 versus System 2 processing produce a similar effect. If the affect heuristic in risk and benefit judgments is indeed primarily a System 1 process, we hypothesize that we could relate the individual (inverse) correlation coefficient (i.e., an index of affect heuristic) to individual cognitive abilities. In Study 2 of this project, we administer an extensive test battery tapping various cognitive abilities, such as executive functions and working memory ability as well as measures of cognitive reflection, numeracy, and risk literacy. Thus, a novel contribution of the current research would be to link the propensity to use the affect heuristic to individual cognitive abilities. Although tests of cognitive abilities, such as working memory capacity or executive functions, such as inhibition of distracting elements, are tapping performance on various System 2 processes, it remains an open question as to whether these abilities relate to the propensity of using System 1 procedures. By looking at different facets of cognitive abilities, we can get a better understanding of the mechanisms that may explain why some individuals may or may not utilize the affect heuristic. For example, it does not necessarily follow that someone with superior attention span (i.e., executive functions) is more apt at overriding or bypassing System 1 processes in favor of more controlled and perhaps rational cognitive processes. On the other hand, it could very well turn out that superior cognitive abilities lead to more deliberate evaluations of risk and benefits. An individual may identify an affective response toward a choice in a decision-making context but be able to override the gut feeling in favor of an evaluation made in a more deliberate state. Yet another example could be if we find a link between less reliance on the affect heuristic and working memory capacity. If so, one might surmise that the mechanism would be inherently different than if, say, inhibition capability was the defining feature. If working memory is a determinant, this might be so perhaps because of a limited mental workspace capacity to carry out mental computation of risk and benefit as separate entities. Inhibition may explain it differently by inhibiting intuitive, affective, or irrelevant responses that come to mind when evaluating risk and benefit. These are two examples of how two different System 2 processes can explain the propensity to use the affect heuristic but with different underlying mechanisms. To investigate this possibility, we administered a set of tests tapping general cognitive abilities that could plausibly be tied to the propensity to use the affect heuristic.

Besides the traditional cognitive abilities described above, other measures have been used to investigate System 2 processes. Cognitive reflection is the mechanism by which intuitive errors are identified and overridden, and scores on the three-item Cognitive Reflection Test (CRT) have been linked to normative decision making (Frederick, 2005). Scores on the CRT have been linked to risk preferences (Frederick, 2005), but no study has yet to investigate the link to risk and benefit judgments. Individuals high on cognitive reflection may be less inclined to exploit the affect heuristic and instead be more able to evaluate risks and benefits in a deliberate state.

Another instrument that is associated with normative decision making is the Berlin Numeracy Test (BNT; Cokely et al., 2012), which is a measure of statistical numeracy and risk literacy. According to the developers, the BNT captures a skill that is “…important for accurately interpreting and acting on information about risk—i.e., risk literacy” (Cokely et al., 2012, p. 37). If the BNT measures individual numeracy and risk literacy, it is likely that these individuals would make more normative decisions of risk judgments. A plausible hypothesis is that higher risk literacy results in less propensity to use the affect heuristic (see also Ikawa and Kusumi, 2018). It should, therefore, result in a weaker inverse relationship between risk and benefit judgments.

### Aims of the Present Research

We examine whether the affect heuristic in risk judgment can be captured using activities from various different domains (e.g., social domain, sensation-seeking domain, health domain, economic domain, etc.) and whether the affect heuristic is sensitive to elicitation method effects (joint/separate evaluation). Although individual analyses of the different domains are outside the scope of the current study, using a large questionnaire with a variety of everyday activities that are not necessarily infused with strong affect (as opposed to studies investigating attitude toward nuclear power plants or biotechnology) would strengthen the notion that the affect heuristic is involved ubiquitously in everyday judgments of risk and benefits. By letting independent groups fill out separate questionnaires of risk and benefit judgments and comparing them to a third group that makes joint evaluations of risk and benefit, we assess the stability of the affect heuristic across elicitation methods.

In Study 2, we go deeper to investigate individual cognitive abilities involving System 2 processes that may drive the affect heuristic. Here, we will investigate individual slopes of risk and benefit judgments and compare them to individual cognitive abilities. The individual slope (correlation coefficient) would constitute an index of whether an individual relies on the affect heuristic. Is the propensity to use the affect heuristic in risk and benefit judgments linked to specific cognitive abilities? We administer a test battery of standard cognitive abilities, such as general intelligence, executive functions, and working memory. In addition, we investigate numeracy and risk literacy as measured by the BNT and CRT that has been explicitly linked to System 1 and System 2 processes. We expect that the BNT and the CRT will be linked to the propensity to use the affect heuristic.

## Study 1: Establishing the Affect Heuristic

To verify the stability of the involvement of the affect heuristic in risk and benefit judgments, we developed a questionnaire (see brief description below). If the judgments of risk and benefit are sensitive to whether they are made in joint or separate evaluation, we would expect a difference in the strength of the correlation coefficient between conditions. If, on the other hand, the correlation coefficients are similar between joint evaluations and separate evaluations, then the stability of the affect heuristic across elicitation methods is supported.

### Method

#### Participants

An online survey (described below in Section “Material” of Study 1) administered by CMA Research was created and sent out to a sample of 602 Swedish adults aged 19–35 (328 women, 269 men, and 5 unspecified). The mean age in the sample was 28.08 years (*SD* = 4.23). Each individual was randomly assigned to one of three groups: (1) Risk-Only (RO), (2) Benefit-Only (BO), or (3) Risk–Benefit (RB). The RO (*N* = 204) group was only asked to fill out the form and rate each activity based on the perceived level of risk. The BO (*N* = 202) group filled out the same questionnaire but was instructed to rate each activity based on the level of perceived benefit. The RB group (*N* = 196) filled out both questionnaires in a counterbalanced design. Thus, half the RB group started with the risk questionnaire, whereas the other half started with the benefit questionnaire.

Upon clicking the link to the survey, the participants first entered their age and gender before being presented with the instruction screen. The participants then completed the 64-item questionnaire if being assigned to a separate condition or both 64-item questionnaires if being assigned to the joint RB condition. After completing the questionnaire in its entirety, the participants were paid $5.

#### Material

To investigate the affect heuristic, we developed a questionnaire containing 64 items. These items consisted of various activities in different domains. They were adapted from previous sources investigating risk perceptions and risky behavior (Slovic, 1987; Weber et al., 2002) and from Bradley and Lang (1999). The domains from which the risky activities were selected included the social domain (e.g., “Speak before an audience,” “Having an affair”), the health domain (e.g., “Undergo surgery,” “Vaccination”), the sensation-seeking domain (e.g., “Skydiving,” “Taking ecstasy”), the economic domain (e.g., “Buy stocks,” “Housing mortgage loan”). We also included more leisurely, low-risk, everyday activities in the same domains (e.g., “Play chess,” “Read a book”) as well as more medium-risk activities (e.g., “Horseback riding,” “Ice skating on a frozen lake”). The items were pseudo-randomly distributed throughout the questionnaire, and the participants were asked to rate each activity based on his/her subjective attitude from 1 (not at all risky/beneficial) to 7 (extremely risky/beneficial). There was no time pressure to complete the questionnaire.

### Results

**FIGURE 1:**
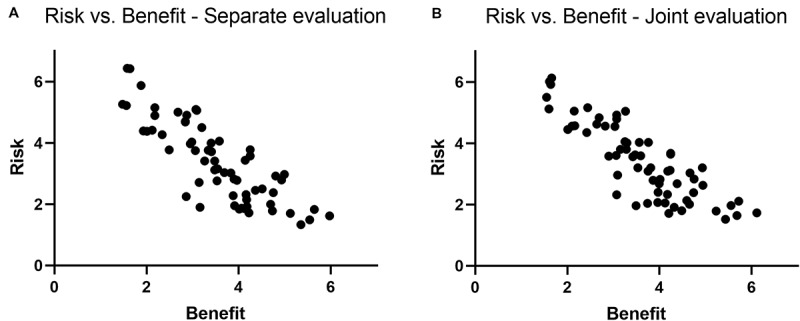
Scatterplot of the relationship between risk and benefit judgments in **(A)** separate evaluation and in panel **(B)** joint evaluation.

Prior to making the main analyses, we performed quality control by looking at respondents’ answers and excluding conspicuous instances of respondents whose response patterns were invalid (e.g., respondents who only rated 1s throughout the entire questionnaire). Twenty-seven participants were excluded from further analyses, resulting in a final sample of *N* = 575 (RO = 195, BO = 193, RB = 187). For each group, all items were averaged with respect to their perceived level of risk or benefit. The correlation between judged risk and judged benefit across the 64 items was then calculated for the joint RB-group as well as for the separate RO and BO groups. The group-level correlation for the RB group was *r* = −0.85, *p* < 0.001, and for the separate RO–BO group it was *r* = −0.86, *p* < 0.001. See Figure 1 for a scatterplot of both joint and separate evaluations. We investigated whether judgments of risk and benefit were the same for the separate and joint conditions by using a test of statistical equivalence (TOST) using a smallest effect size of interest (SESOI) of one half of a standard deviation of the mean risk ratings and benefit ratings. The test showed the risk ratings were statistically equivalent, *t*(126) = 2.57, *p* = 0.006) irrespective of whether they were evaluated separately or jointly. Judgment of benefit was also equivalent between the RB-group and the RO–BO group, *t*(126) = 2.54, *p* = 0.006. To further investigate the stability of the correlations, we calculated rank-order correlations for the groups, but the correlation coefficients remained the same, except for the RB-group that dropped from *r* = −0.85, *p* < 0.001 to *r*_s_ = −0.80, *p* < 0.001. To see whether the negative correlation was prevalent across domains, we calculated correlation coefficients for the activities within each domain. The 64 activities were divided into four domains (health, sensation-seeking, social/economic, and recreation), and the domain-specific correlations were all negative. Strong inverse correlations were observed for all domains except the recreation domain. Specifically, the social/economic (*r* = −0.82), health (*r* = −0.92), and sensation-seeking (*r* = −0.76) were strong, whereas the recreation domain showed weaker correlation (*r* = −0.35), which is plausible given that many recreational activities involved both very little obvious risk and benefit (e.g., watching TV, playing chess). We also calculated each individual’s risk and benefit ratings across the 64 items to establish an individual correlation coefficient. This correlation coefficient can be construed as an index (risk–benefit index; RBI) of individual inclination to use the affect heuristic. For the RB group, the mean correlation was −0.50 (*SD* = 0.33).

### Discussion

The findings based on the RB group, who filled out both risk and benefit judgments in a joint evaluation, showed an inverse correlation of *r* = −0.85, *p* < 0.001, echoes previous studies by Finucane et al. (2000), illustrating that our questionnaire captures the affect heuristic. By looking at the separate evaluations and looking at the correlations between risk and benefit judgments of the activities, we find an almost identical pattern (*r* = −0.86, *p* < 0.001). This indicates that participants likely used the affect heuristic across all conditions and used affect as an index of the relative risk and benefits of these activities. Still, given that this measurement is performed on the average group level, one should be wary of making strong conclusions about individual-level mechanisms that drive these response patterns. However, we find plausible support for the stability of the affect heuristic in risk and benefit judgments, irrespective of whether the judgments are made separately or jointly.

## Study 2: the Affect Heuristic and Individual Cognitive Abilities

In the second study, we sought to explore the potential relationship between the tendency to use the affect heuristic in risk and benefit judgments and individual cognitive abilities. Given that the affect heuristic may be considered as a System 1–driven process, it could very well be negatively tied to cognitive processing abilities, such as logical reasoning, executive functions, numeracy and risk literacy, and cognitive reflection. To this end, we recruited a sample of university students that underwent testing of a cognitive test battery as well as the risk–benefit questionnaire. Finucane et al. (2000) found that time pressure induced a mode in which participants were more likely to resort to a quick and intuitive mode of thinking that, in turn, led the participants to rely even more on the affect heuristic. Here, we do not explicitly manipulate the modes in which risk and benefit judgments are made (cf. Finucane et al., 2000; Keller et al., 2006), but rather look at individual differences pertaining to System 2 capacities and whether there is a link between cognitive abilities and the inclination to use the affect heuristic. Although this is primarily an explorative study, we suspect that certain cognitive dispositions, say, high cognitive reflection ability, will allow individuals to utilize cognitive resources to reflect upon the risk and benefits judgments to be made. This may allow individuals to identify a potential discrepancy between gut feelings about a certain event and the more objective features of those events that may be identified upon reflection. If so, we would expect that individuals with better cognitive abilities, which are dependent upon System 2 processes, would display a weaker or even absent inverse correlation between risk and benefit judgments. Conversely, poorer cognitive reflection scores or other System 2–driven abilities likely indicate that an individual relies on affective markers with which the activities or scenarios are tagged. Accordingly, poorer performance on these tasks would be associated with stronger inverse correlations between judgments of risk and benefit. We administer a cognitive test battery to explore this potential relationship. We are primarily interested in established general cognitive abilities (e.g., executive functions, working memory, and spatial ability), numeracy and risk literacy, and cognitive reflection. These abilities may be involved in attending to relevant information and inhibiting distracting elements. Math performance was also assessed as a way to investigate whether explicit calculation efficiency was linked to individual use of affect heuristic. In addition, we used a measure of general intelligence to primarily control for abstract reasoning when investigating the role of numeracy and risk literacy, executive functions, and cognitive reflection in relation to the affect heuristic.

### Method

#### Participants

The sample consisted of 41 participants (21 males, 20 females) recruited from Linköping University. The mean age of the sample was 23.29 (*SD* = 3.08). The participants were recruited from different faculties; 21 of the participants were enrolled at the faculty of arts and science, and 20 were recruited from the technical faculty. All participants had normal or corrected-to-normal vision. Participants with a history of neurologically based impairments, such as ADHD or other known learning disabilities (e.g., dyslexia and dyscalculia) were excluded. All participants gave their informed and written consent, and the study was approved by the local ethics committee.

The testing was divided into three separate sessions. In the first session, the participants completed the numeracy test and CRT. The second session contained Raven’s Advanced Progressive Matrices (RAPM; Raven, 2000), arithmetic calculation, mental rotation, and the executive function tasks (digit span, shifting, inhibition). In the third and final session, the participants completed the risk–benefit questionnaire. All testing was completed within 4 months. Instructions were read aloud by an experimenter from a printed manuscript, and all tests were administered in the same order for all study participants. Computer-based tasks were run on a laptop, using SuperLab PRO 4.5.

#### Material

##### General intelligence

We measured general intelligence using a short version of RAPM developed and normed by Bors and Stokes (1998). The short version contains 12 items taken from the original RAPM that have proven to be a useful and valid proxy for the full-length RAPM (*r* = 0.92 correlation with full RAPM; Bors and Stokes, 1998). Each test item contained a figure or matrix with a set of elements that together complete a logical pattern involving both horizontal and vertical transformations. For each test item, there is one missing piece of the figure. The participant has to infer which figure would complete the pattern out of eight alternatives. The participants had 20 min at their disposal to solve as many problems as possible.

##### Executive functions – shifting and inhibition

To assess executive functions, we administered a shifting task (trail-making test) and an inhibition task (Stroop task). The trail-making test was in paper-and-pencil format and contained 22 circles, each with a digit or a letter. The participants are told to draw a line and connect the circles in ascending an alternating order (1-A-2-B-3-C, etc.) as quickly as possible. Seconds to complete each condition was used as the dependent measure.

The Stroop task consisted of two sheets of paper containing 30 written color words divided into two columns on each sheet. The color in which the words were written and the color the words signified were incongruent (e.g., the word “blue” written in red letters). The participant was told to verbalize the color in which the words were written as quickly as possible while inhibiting the meaning of the words. Each sheet was completed separately, and the time it took for the participants to name all 30 colors on a sheet was used as the dependent measure. The mean response time of the two sheets was used as the index of inhibition ability.

##### Executive functions – working memory

The digit span subtest of the Wechsler Adult Intelligence Scale IV (WAIS-IV; Wechsler, 2011) was used to measure working memory ability. This subtest contains three conditions: digit span forward, digit span backward, and digit span sequencing. In the digit span forward condition, the participant hears a series of digits and attempts to repeat them out loud in order. In the digit span backward condition, the participant has to repeat the string of digits in reverse order. The sequencing condition requires the participant to recall all the digits in the correct ordinal sequence. All conditions become increasingly more difficult in terms of the number of digits there are to be repeated. The maximum score for each condition is 16 for a total of 48 points max.

##### Cognitive reflection

We administered the CRT (Frederick, 2005) containing three items to measure cognitive reflection. The phrasing of the problems is constructed in such a way that intuitive but wrong solutions have to be inhibited. The following questions are part of the CRT: (1) “A bat and a ball cost $1.10. The bat costs $1.00 more than the ball. How much does the ball cost?” (2) “If it takes five machines 5 min to make five widgets, how long would it take 100 machines to make 100 widgets?” (3) “In a lake, there is a patch of lily pads. Every day, the patch doubles in size. If it takes 48 days for the patch to cover the entire lake, how long would it take for the patch to cover half of the lake?” The number of correctly answered problems was used as an index of cognitive reflection ability.

##### Visuospatial ability

Visuospatial ability was measured using a paper-and-pencil mental rotation test. This test consisted of 16 items in the form of cube figures. A reference figure was located on the left side, and four comparison figures were located to the right of the reference figure. Two “correct” and two mirrored items were illustrated as comparison items. The task was to identify the two matching figures and subsequently mark them with a pen. The comparison stimuli were rotated in the picture-plane in one of either six rotation angles: 45°, 90°, 135°, 225°, 270°, or 315°. The participants had 4 min to solve as many problems as possible. Both correct comparison figures needed to be marked in order to obtain one point for the item, yielding a maximum score of 16.

##### Numeracy and risk literacy

Numeracy and risk literacy was measured using the BNT, developed by Cokely et al. (2012). The scores from the BNT have been found to be normally distributed in an educated population. The BNT consists of four items (e.g., “Out of 1000 people in a small town, 500 are members of a choir. Out of these 500 members in the choir, 100 are men. Out of the 500 inhabitants that are not in the choir, 300 are men. What is the probability that a randomly drawn man is a member of the choir?). The BNT can be administered in an adaptive format, requiring the participants to solve only three problems in quick administration time. However, we chose to use all four items of the scale and aggregate all correct answers as an index of numeracy and risk literacy, which is a valid alternative (Cokely et al., 2012).

##### Arithmetic calculation

Arithmetic ability was measured using four subtests (addition, subtraction, multiplication, and division) using a similar procedure as Gebuis and van der Smagt (2011). This paper-and-pencil test contained arithmetic problems of increasing difficulty (e.g., “34 + 12” and “67 + 76” in the addition subtest and “8 × 13” and “62 × 8” in the multiplication subtest). The participants were instructed to complete as many problems as they could within the allotted time of 120 s for each subtest. The difficulty level of the problems was manipulated by increasing the number of digits or by requiring borrowing or carrying. Each subtest contained 54 problems except for division, which contained 26. The total number of correctly solved problems across all four conditions was used as an index of arithmetic ability.

##### Risk–benefit questionnaire

This questionnaire was almost equivalent to the one used in Study 1. However, there were two differences. First, the questionnaire was filled in with a pen and paper instead of on a computer. Second, the participants filled in the questionnaire in two steps. The first step was equivalent to Study 1, but the second step included an opportunity to adjust one’s judgments when having a definition next to the activities. This was primarily used to investigate the degree to which participants interpreted the activities as intended. Only minor changes were made by some participants, and we concluded that the questionnaire, and the activities therein, are interpreted as intended when using internet surveys of this questionnaire. Below are the judgments made by the participants after introducing the definitions is used for analysis.

### Results

**FIGURE 2:**
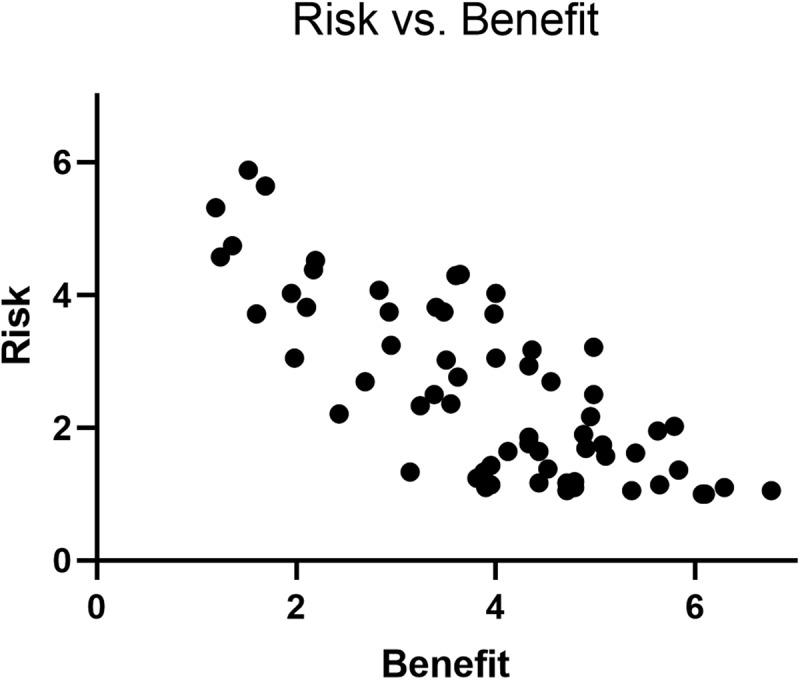
Scatterplot of the relationship between risk and benefit judgments in Study 2.

The risk and benefit judgments of all activities can be found in Table 1. An overview of the descriptive results and a correlation matrix can be found in Table 2. In the scatterplot in Figure 2, we observe the same overall pattern as in Study 1 in terms of the risk–benefit correlation. Mean risk and benefit judgments across the 64 situations show a strong correlation, *r* = −0.77, *p* < 0.001. Calculating a rank-order correlation revealed a slight decrease in the coefficient, *r_s_* = −0.73, *p* < 0.001. As in Study 1, the pattern of negative correlations for activities in each domain showed similar patterns. Activities in the social/economic domain showed a correlation of *r* = −0.80, *p* = 0.029; the health domain *r* = −0.86, *p* = 0.001; the sensation-seeking domain *r* = −0.65, *p* = 0.007; and the recreation domain *r* = −0.33, *p* = 0.180. Furthermore, all participants except three had a significant negative intra-individual correlation between their risk and benefit judgments. All intra-individual correlations, significant and non-significant together, have a mean of −0.54 (*SD* = 0.17) with a range between −0.04 and −0.86. This suggests, as in Study 1, that some have stronger negative linearity than others. It is worth noting that the standard deviation within this group is roughly half the size than for the groups of Study 1.

**TABLE 1:** Risk and benefit judgments in Study 2 sorted by level of estimated risk.

		**Risk**	**Benefit**			**Risk**	**Benefit**			**Risk**	**Benefit**
	**Activity**	***M***	***M***		**Activity**	***M***	***M***		**Activity**	***M***	***M***
1.	Take ecstasy	6.28	1.65	23.	Ice skating on a lake	3.88	3.32	45.	Shopping	2.49	4.08
2.	Take cocaine	6.22	1.59	24.	Snowboarding	3.77	3.17	46.	Leave blood	2.39	4.74
3.	Smoking	5.89	1.76	25.	Have kids	3.73	4.24	47.	Bowling	2.34	3.93
4.	Shoplifting	5.38	1.51	26.	Get divorced	3.68	2.69	48.	Play golf	2.28	2.97
5.	Cheating on partner	5.17	1.58	27.	Go skiing	3.67	3.44	49.	Drink coffee	2.24	4.17
6.	Handling guns	5.15	2.31	28.	Drive a car	3.60	4.25	50.	Eat chocolate	2.07	4.64
7.	Mountaineering	5.07	3.17	29.	Eat sugar	3.51	3.16	51.	Play video games	2.00	3.83
8.	Speeding with a car	4.97	2.17	30.	Horseback riding	3.50	3.53	52.	Watch TV	1.99	4.15
9.	Skydiving	4.93	3.08	31.	Eat red meat	3.34	3.45	53.	Take a walk	1.97	5.68
10.	White water rafting	4.91	2.97	32.	Switch career	3.28	4.18	54.	Drink juice	1.96	4.01
11.	Bungee jumping	4.78	2.75	33.	Take painkillers	3.18	3.53	55.	Play chess	1.93	3.32
12.	Unprotected sex	4.77	2.77	34.	Rollercoaster	3.11	3.82	56.	Eat dinner	1.91	5.60
13.	Drink strong spirits	4.65	2.74	35.	Bicycling	3.09	4.96	57.	Eat an apple	1.90	4.69
14.	Drink alcohol	4.52	3.12	36.	Fly commercially	3.06	3.72	58.	Yoga	1.87	4.16
15.	Casino gambling	4.49	2.17	37.	Swimming	2.97	4.73	59.	Play board games	1.79	4.16
16.	Snuffing tobacco	4.47	2.02	38.	Have an X-ray	2.95	4.04	60.	Drink tea	1.76	4.35
17.	Online Casino	4.42	2.01	39.	Buy scratch tickets	2.83	3.12	61.	Eat a salad	1.74	5.17
18.	Sun tanning (salon)	4.31	2.38	40.	Vaccinating	2.81	4.84	62.	Drink water	1.67	6.04
19.	Buy stocks	4.04	3.67	41.	Hold a speech	2.79	3.77	63.	Resting	1.56	5.39
20.	Wave surfing	4.04	3.11	42.	Go by ferry	2.79	3.91	64.	Reading	1.42	6.10
21.	Skiing in the Alps	4.01	3.48	43.	Go by train	2.59	4.45				
22.	Take a bank loan	3.89	3.05	44.	Jogging	2.54	4.65				

**TABLE 2:** Descriptive data and correlation matrix.

	**Measurements**	**Mean (SD)**	**1**	**2**	**3**	**4**	**5**	**6**	**7**	**8**	**9**
1.	General intelligence	8.90 (2.7)	–	0.42**	–0.22	–0.05	0.44**	0.46**	0.67**	0.51**	0.47**
2.	Visuospatial ability	7.32 (4.03)	0.42**	–	–0.22	0.05	0.43**	0.45**	0.42**	0.37*	0.23
3.	EF – shifting	40.82 (15.24)	–0.22	–0.22	–	0.35*	−0.33*	–0.24	–0.08	−0.50**	–0.07
4.	EF – inhibition	25.70 (4.35)	–0.05	0.05	0.35*	–	0.09	–0.02	0.01	−0.32*	0.01
5.	Working memory	26.59 (5.21)	0.44**	0.43**	−0.33*	0.09	–	0.48**	0.60**	0.56**	0.11
6.	Cognitive reflection	1.78 (1.06)	0.46**	0.45**	–0.24	–0.04	0.48**	–	0.48**	0.51**	0.44**
7.	Num./risk literacy	2.24 (1.24)	0.67**	0.42**	–0.08	0.01	0.60**	0.48**	–	0.66**	0.44*
8.	Arithmetic ability	107.59 (20.22)	0.51**	0.37*	−0.50**	−0.32*	0.56**	0.51**	0.66**	–	0.36*
9.	Risk/benefit *r*	−0.54(0.17)	0.47**	0.23	–0.07	0.01	0.11	0.44**	0.41*	0.36*	–

*Correlation is significant at the 0.05 level (two-tailed). **Correlation is significant at the 0.01 level (two-tailed).

**FIGURE 3:**
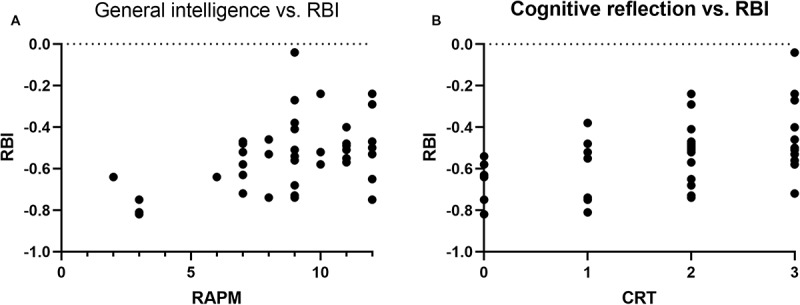
Scatterplots of the relationship between individual RBI and **(A)** general intelligence and **(B)** cognitive reflection.

When looking at the correlations between the various measures and the risk–benefit index (RBI), we find that RBI correlates with general intelligence (*r* = 0.47, *p* < 0.01), CRT (*r* = 0.44, *p* < 0.01), numeracy/risk literacy (*r* = 0.44, *p* < 0.01), and arithmetic ability (*r* = 0.36, *p* < 0.01). To investigate the relationship between these measures and how they relate to RBI, we calculated partial correlations with RAPM as a covariate. Numeracy, CRT, and arithmetic are arguably dependent on logical reasoning skills; thus, we controlled for RAPM to see whether numeracy, CRT, and arithmetic could still explain unique variance. The partial correlation, controlling for RAPM, showed that the correlations between RBI, numeracy, and arithmetic disappeared. However, the correlation between RBI and CRT remained (*r* = 0.32, *p* = *0.043*). Thus, there is indeed a relationship between RBI and cognitive reflection but not between RBI and numeracy and risk literacy once intelligence is taken into account. See Figure 3 for scatterplots of the relationships between RBI, CRT, and general intelligence.

### Discussion

The findings from Study 2 indicate that whether one uses the affect heuristic in risk judgments may depend on individual cognitive abilities. Although maintaining an explorative stance, we expected that several general cognitive abilities would be related to risk and benefit judgments. Still, the only abilities that were linked to individual RBI were general intelligence, arithmetic performance, numeracy/risk literacy, and cognitive reflection. Executive functions, spatial ability, and working memory capacity did not link to RBI although cognitive reflection did even when controlling for general intelligence. Established measures of general cognitive abilities are inherently about mental capacities although cognitive reflection may also involve a general tendency or inclination to identify and resists responses that first come to mind (Frederick, 2005). The affect heuristic is closely linked to the availability heuristic (Tversky and Kahneman, 1982), and it has been suggested that the affect heuristic is essentially a type of availability process in which emotionally charged events quickly spring to mind (Slovic et al., 2004). Therefore, individuals who perform less well on the CRT may not tend to inhibit these affective or intuitive responses and, thus, act according to their intuitive gut feelings when judging risks and benefits. A study by Thoma et al. (2015) found that professional financial traders showed higher CRT scores than non-trading bank employees and individuals outside the world of finance. Moreover, traders also displayed higher risk-taking behavior than the other groups, which could suggest a link between cognitive reflection and the inclination to take risks despite possibly negative emotional reactions that accompany those risks. Thus, individuals high in cognitive reflection may be able to override initial affective reactions to different contingencies or events and instead make risk assessments in a more deliberate state.

Interestingly, numeracy and risk literacy did not relate to the affect heuristic once intelligence was controlled for. So even if the BNT measures numeracy and risk literacy, it does not appear to have a specific and strong impact when judging the amount of risk a given activity entails above and beyond intelligence. Nevertheless, performance on numeracy and risk literacy measurements likely tap into the ability to process and solve problems concerning risk when numerical information is pertinent to the situation at hand. Taken together, our findings indicate that the tendency to use the affect heuristic (RBI), on a group level, does relate to a specific cognitive ability, namely the ability or disposition to identify and resist responses that first comes to mind.

## General Discussion

The overall aim of this study was to investigate the stability of the affect heuristic, both in terms of methodological elicitation (joint vs. separate evaluation) and in terms of cognitive abilities. The finding from Study 1 establishes that the affect heuristic in risk judgments is indeed a robust phenomenon that is reproducible in both joint and separate conditions. This is important because research has shown that people make different evaluations about preferences depending on whether the options are presented in isolation or not (Hsee, 1996; Hsee et al., 1999). However, our results imply that the inverse relationship can be elicited irrespective of whether the judgments of the relative risks and benefits are made jointly or separately. This reinforces the robustness of the affect heuristic as a phenomenon when making judgments of risk and benefits.

By developing a questionnaire containing activities from various different domains and levels of risk, we could also generalize the prevalence of the affect heuristic to not only include highly salient phenomena events such as nuclear power, climate change, or biotechnology. Thus, the affect heuristic is a ubiquitous feature of everyday life when judging risks and benefits. In addition, we find that the affect heuristic can be indexed on an individual level. In Study 2, we find that this affect heuristic index can be tied to individual cognitive abilities, primarily cognitive reflection ability. This corroborates previous work by Finucane et al. (2000) that demonstrated that the inverse relationship between perceived risks and benefits increased greatly under time pressure, when the opportunity for analytic deliberation was reduced. Thus, the inverse relationship between risk and benefit judgments may be driven by System 1 processes, which our findings support. The ability to inhibit System 1 impulses or intuitions, as measured by the CRT, is, thus, related to whether one relies on the affect heuristic or not.

Although we administered a comprehensive test battery of well-established cognitive measurements, we failed to find a link between executive functions or working memory and the tendency to use the affect heuristic. *Prima facie*, executive functions and working memory capacity would plausibly be associated with the affect heuristic insofar as having poor cognitive capacities may undermine the ability to reflect deliberately and disregard discrepant affective reactions during judgments of risks and benefits. Despite the apparent correlation between working memory and CRT, only CRT correlated with the affect heuristic index when controlling for intelligence. Numeracy and risk literacy was associated with the affect heuristic, but the relationship disappeared once we controlled for intelligence, suggesting that the apparent link was likely attributed to abstract reasoning and logic rather than a specific capability to process probabilities and risk information. Still, this does not entail that numeracy and risk literacy is unimportant during risk judgments overall. It likely is important in such judgments. But numeracy and risk literacy appears not to predict whether one uses the affect heuristic during risk and benefit judgments once logical reasoning ability is accounted for.

The nature of the interactions between System 2 and System 1 processes are important to investigate, and there could be multiple potential pathways through which these mechanisms could be working. Our current study, in which we measure cognitive performance, could be regarded as targeting the “algorithmic mind” of Stanovich’s (2011) tripartite model of the mind. According to this model, there are three modes of thinking, two of which correspond to System 2 processing (“the algorithmic mind” and “the reflective mind”), and one corresponds to System 1 processing (“the autonomous mind”). The algorithmic mind is the level at which individual cognitive performance takes place (e.g., working memory processing and fluid intelligence), whereas the reflective mind refers to individual differences in rational thinking dispositions. Thinking dispositions, such as “need for cognition” (e.g., Epstein et al., 1996) or “lay rationality” (Hsee et al., 2015) are undoubtedly influential determinants of whether one engages in various heuristics and biases. One might, therefore, contrast “disposition” versus “ability,” and both are surely important contributors to decision making. Therefore, future studies should employ comprehensive test batteries in which both performance measures as well as measures of individual dispositions are included to get a full picture of how, when, and by whom the affect heuristic is used.

The results obtained from these studies should also be explored in more detail in future follow-up studies. Given the small sample in Study 2, our correlations and partial correlations should be interpreted with caution. The approach at the outset was mainly exploratory as we employed a broad set of established cognitive tests, and the results should be verified more rigorously. Still, a strength of Study 2 was that all testing was supervised and strictly controlled, which is also necessary when employing standardized cognitive tests. A methodological strength is that we could find an almost identical pattern when administering the risk–benefit questionnaire online to 600 participants as when we administered it individually in a closely supervised setting. Thus, the results are promising, both in terms of the stability of the affect heuristic in a supervised versus non-supervised setting and also in that it is stable across separate and joint evaluation conditions.

## Data Availability Statement

The data sets generated for this study are available on request to the corresponding author or through Open Science Foundation website (https://osf.io/7tpf4/quickfiles).

## Ethics Statement

Ethical review and approval was not required for the study on human participants in accordance with the local legislation and institutional requirements. The patients/participants provided their written informed consent to participate in this study.

## Author Contributions

KS and DV together conceptualized the study and contributed to the study design. MF collected data and performed data analysis. KS interpreted the results and drafted the manuscript. PS and DV contributed with interpretations and revisions of the manuscript draft. All authors approved the final version of the manuscript for submission.

## Conflict of Interest

The authors declare that the research was conducted in the absence of any commercial or financial relationships that could be construed as a potential conflict of interest.
